# Is there sufficient evidence to support the health benefits of including donkey milk in the diet?

**DOI:** 10.3389/fnut.2024.1404998

**Published:** 2024-09-25

**Authors:** Muhammad Zahoor Khan, Wenting Chen, Mengmeng Li, Wei Ren, Bingjian Huang, Xiyan Kou, Qudrat Ullah, Lin Wei, Tongtong Wang, Adnan Khan, Zhenwei Zhang, Liangliang Li, Changfa Wang

**Affiliations:** ^1^Liaocheng Research Institute of Donkey High-Efficiency Breeding and Ecological Feeding, Liaocheng University, Liaocheng, China; ^2^Department of Theriogenology, Faculty of Veterinary and Animal Sciences, Cholistan University of Veterinary and Animal Sciences, Bahawalpur, Punjab, Pakistan; ^3^Genome Analysis Laboratory of the Ministry of Agriculture, Agricultural Genomics Institute at Shenzhen, Chinese Academy of Agricultural Sciences, Shenzhen, China

**Keywords:** donkey milk, lysozyme, lactoferrin, antioxidant, antibacterial, immune regulator, therapeutic potential

## Abstract

Donkey milk has attracted attention due to its distinctive nutritional composition and potential health advantages, particularly because of its whey protein content, which includes lysozyme, *α*-lactalbumin, lactoferrin, and *β*-lactoglobulin and vitamin C, among other components. These elements contribute to immunoregulatory, antimicrobial, antioxidant, and anti-inflammatory properties, positioning donkey milk as a possible therapeutic option. In addition, due to the low levels of caseins, the casein-to-whey protein ratio, and the *β*-lactoglobulin content in donkey milk, it presents an optimal alternative for infant formula for individuals with cow’s milk allergies. Moreover, research into donkey milk’s potential for cancer prevention, diabetes management, and as a treatment for various diseases is ongoing, thanks to its bioactive peptides and components. Nevertheless, challenges such as its low production yield and the not fully understood mechanisms behind its potential therapeutic role necessitate more thorough investigation. This review consolidates the existing knowledge on the therapeutic possibilities of donkey milk, emphasizing its importance for human health and the need for more detailed studies to confirm its health benefits.

## 1 Introduction

The therapeutic potential, similarities to human milk and high digestibility of donkey milk have captured the attention of researchers in this field (1–4). Donkey milk has low cholesterol, fat and protein level, however, consider a rich source of milk whey proteins (3, 5). In addition, donkey milk has a vitamin C level of 57 mg/L, which is closest to that of human milk (60 mg/L), and higher than cow milk (27 mg/L) (1, 6). The low level of casein and casein to whey protein ratio in donkey milk may contribute to its role in formula milk of infant (3, 7). Today, donkey milk is being marketed as a consumer product and is used by newborns (8, 9), people with cow’s milk protein allergies, and the elderly (10).

The growing interest in donkey milk is further justified by its multifaceted bioactivities, encompassing antimicrobial, antiviral, anti-inflammatory, anti-proliferative (2, 11–13) and antioxidant effects (3, 14). Additionally, its pronounced antibacterial activity against a spectrum of pathogenic bacteria (2) is linked to an extended shelf life of the milk. The immunomodulatory, anti-inflammatory, and antiviral properties of donkey milk can largely be attributed to its constituent bioactive molecules, namely lactoferrin and lysozyme, as corroborated by extensive research (15, 16).

Lysozyme, a pivotal component of the innate immune system, is recognized for its broad-spectrum antimicrobial activities against bacterial (15), fungal, and viral pathogens, thereby serving as a natural defense mechanism against infections. In the realm of pharmaceuticals, the application of milk-derived lysozyme spans the prophylaxis of diseases of bacterial, viral, fungal, and inflammatory etiologies, further supplemented by its immune-stimulatory (17, 18) and antihistaminic potentials (19, 20). The advancements in lysozyme modification open new avenues in clinical medicine (17, 21). The milk whey proteins were suggested to conduce to the destruction of tumors, as it modulates the synthesis of the tumor necrosis factor (TNFα) and also stimulates the production of Type I interferon (INFα, INFβ, INFγ), interleukin-2 (IL-2) and interleu-kin-6 (IL-6) by human lymphocytes (22).

Based on its abundance in the colostrum and milk, LF plays an essential immunomodulatory role that complements immature biological defense functions in neonates (23). Furthermore, LF enhances immune function, which declines with age (24). The previously reported physiological effects of LF include antimicrobial, antiviral, anti-aging, neuroprotective, regulation of iron metabolism, improvement of bone metabolism, and immunomodulatory effects (25).

The use of donkey milk as a therapeutic agent to heal wounds and treat various diseases, such as bronchitis, asthma, joint pain, and gastritis, is being explored (10, 12). Consistently, the anti-cancer (26), antidiabetics (27), atherosclerosis (28) and anti-colitis (29) properties of donkey milk has also been reported. In the current pandemic of the coronavirus, some modified form of lysozyme can be used to stimulate the formation of interferon, an effective substance against coronavirus, and thus reduce the risk of the life-threatening form of COVID-19 up to 79% (30, 31).

This review aims to synthesize existing knowledge on the antimicrobial, anti-inflammatory, immunoregulatory, and antioxidant attributes of donkey milk. In addition, we have highlighted the role of donkey milk as therapeutic agent in prevention of cancer, diabetes, and heart diseases. Furthermore, this review delineated the principal factors that detrimentally affect the milk characteristics of donkey.

## 2 Materials and methods

This review examines articles that discuss the composition and bioactive components of donkey milk, as well as studies highlighting its potential therapeutic uses. These therapeutic uses include its antimicrobial, antioxidant, anti-inflammatory, and immunomodulatory effects. The data for this review was gathered from reputable platforms such as springerLink, Scopus, Web of Science, PubMed, Google Scholar, and ScienceDirect. Various key terms were utilized in the search, including the composition of donkey milk, its bioactive elements, therapeutic benefits, and its antimicrobial, anti-inflammatory, immunomodulatory, and antioxidant properties. The selection criteria for the articles were rigorous, focusing only on studies published in reputable, peer-reviewed English language journals from 2010 onwards, with the exception of two earlier studies from 2007. Conference summaries, books, and book sections were not considered for this review.

## 3 Composition and bioactive components of donkey milk

The nutritional composition and beneficial properties of donkey milk have been extensively explored in scientific literature, including in recent studies (32–36). The summary of donkey milk compositions has been provided in Table 1. Donkey milk is particularly valued for its close resemblance to human breast milk, both in terms of its nutritional profile and its health benefits. It is characterized by a balanced mix of essential nutrients, though it differs from cow’s milk in several key aspects.

**Table 1:** Comparative analysis of milk composition: donkey, human and cow.

Major components in milk	Composition	Donkey milk	Human milk	Cow milk
Fatty acids (% of total fatty acid)	PUFA (%)	14.0–30.0	8.00–19.00	2.0–6.0
	Linolenic acid (ALA) (%)	4.5–16.00	0.5–3.00	0.5–1.8
	Linoleic acid (LA) (%)	6–15.2	6.0–17.7	1.2–3.0
	LA:ALA (%)	0.9–6.1	7.4–8.1	2.1–3.7
	DHA (%)	0.04	0.15	0.03
	ARA (%)	0.14	0.37	0.48
Major whey protein (g/L)	α-Lactalbumin	1.8–3.0	1.8–3.5	1.0–1.5
	β-lactoglobulin	3.0–3.2	Absent	3.2–4.0
	lysozyme	1	0.04–0.2	Trace
Major casein (CN) (g/L)	β-Casein	3.9	3.8	8.6–11.0
	αs1-Casein	1.2–2.0	1.0–1.9	3.0–3.9
Water (%)		89	87	87
Lactose (g/L)		60–72	63–70	44–58
Protein (g/L)		14–20	9–19	33–40
DM (g/L)		90–114	103–124	118–130
Fat (g/L)		4–16	20–39	34–53
Energy Value (kJ/kg)		1939.4	2855.6	2983.0
Total whey protein (g/L)		4.9–8.0	6.0–8.4	5.0–7.0
Total casein (g/L)		6.5–10.00	2.0–4.2	24.0–29
Casein: whey protein (g/L)		1.2	0.4–0.5	4.5

PUFAs, Polyunsaturated fatty acids; DHA, Docosahesaenoic; ARA, Arachidonic acid.

Protein content in donkey milk ranges from 1.5–2.0%, which is less than that found in cow’s milk. The proteins in donkey milk include both caseins and whey proteins, such as *α*-lactalbumin and *β*-lactoglobulin, but in smaller amounts compared to those in cow’s milk. Notably, donkey milk is richer in lactoferrin and lysozyme, two proteins known for their antimicrobial properties, than cow’s milk (37). Consistently, the fat content in donkey milk is relatively low, between 0.3–1.0%, and its fat includes a higher proportion of polyunsaturated fatty acids, especially linoleic acid, which contributes to its health benefits (38). Donkey milk is also higher in lactose than cow’s milk, with levels between 6.2 and 7.4%, lending it a slightly sweet taste (8).

Moreover, donkey milk is acknowledged for its healthful attributes, such as lower fat and cholesterol levels, and higher amounts of protein, casein, lactose, whey protein, calcium, selenium, and vitamin D3 (36). Consistently another study also reported that donkey milk has a lower cholesterol content, a lower casein to whey protein ratio, a higher calcium to phosphorus ratio, and a higher taurine content compared to bovine milk (39). The donkey milk also contains a variety of vitamins, including both water-soluble (B group and vitamin C) and fat-soluble (vitamins A, D, E, and K) types and minor components such as hormones (6). The vitamin content can fluctuate depending on the donkeys’ diet and environment. Donkey milk is a good source of essential minerals like calcium, magnesium, phosphorus, and potassium, though in amounts typically lower than in cow’s milk (40). Despite this, the bioavailability of these minerals is high, attributed to the low protein content of the milk.

**Figure 1:**
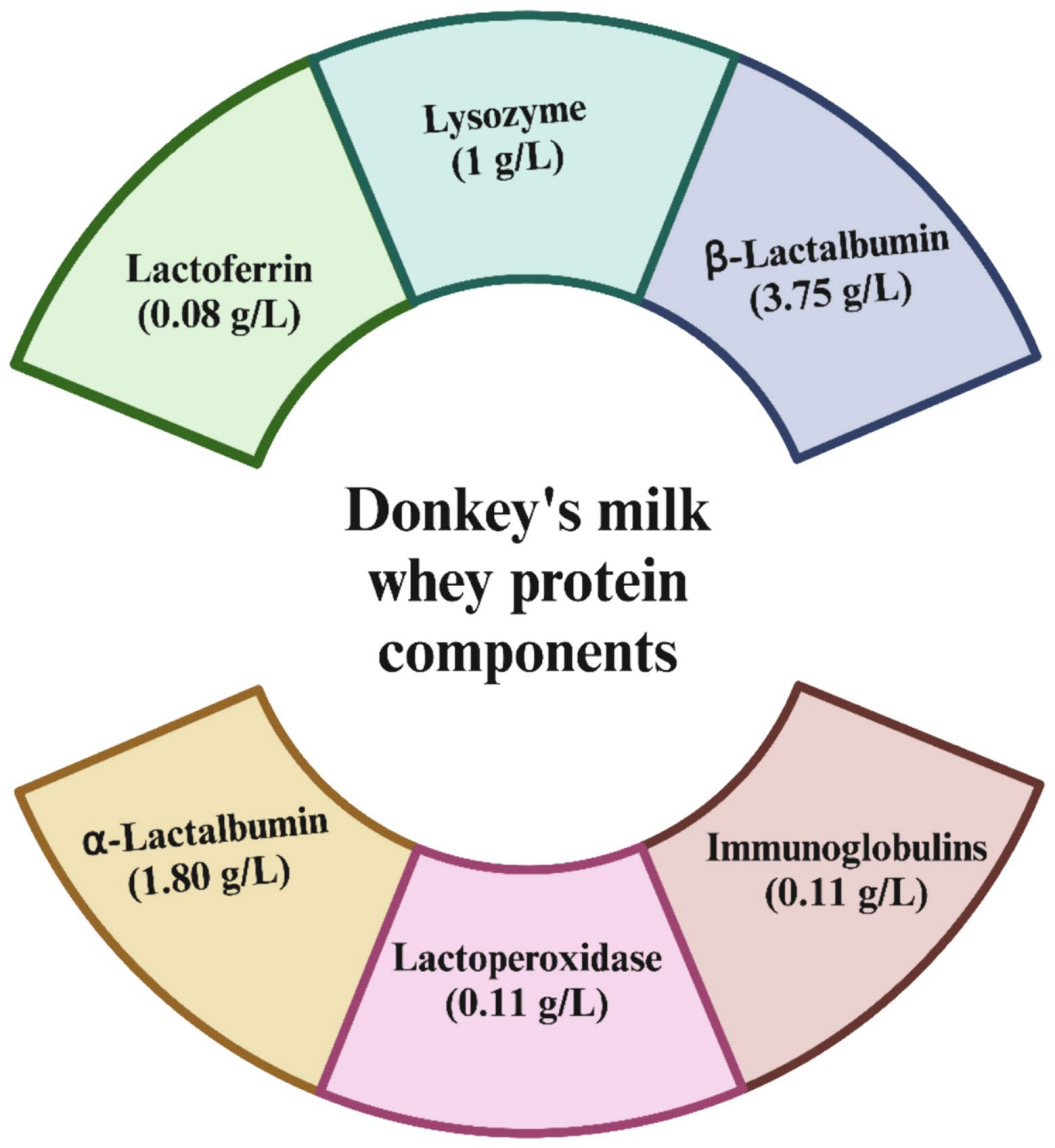
Bioactive components of donkey milk.

Donkey milk also features a range of bioactive components that support immunological health, such as immunoglobulins, lactoferrin, lysozyme, and cytokines, which may enhance its immune-boosting capabilities (41, 42). In terms of whey protein content, donkey milk contains about 4.9–9.6 g/L, which constitutes roughly 43–50% of its total protein. The whey protein is primarily composed of *β*-lactoglobulin, *α*-lactalbumin, immunoglobulins, lysozyme, serum albumin, and lactoferrin (Figure 1). These proteins not only provide energy but also play roles in antimicrobial, antioxidant, anti-inflammatory, and antitumor activities, contributing to the wide range of health benefits associated with donkey milk consumption (43). The whey protein fraction of donkey milk is predominantly constituted by *β*-lactoglobulin, with concentrations approximately at 3.75 g/L, and *α*-lactalbumin, measured around 1.80 g/L. Additionally, the composition includes immunoglobulins at roughly 1.30 g/L, lysozyme at about 1.00 g/L, serum albumin at nearly 0.40 g/L, and lactoferrin at approximately 0.08 g/L (38). After being consumed, proteins are broken down into smaller peptide fragments through enzymatic hydrolysis in the gastrointestinal tract. This process is essential for the efficient digestion and absorption of proteins and also results in the creation of bioactive peptides. These peptides have specific biological functions, often imitating or boosting the activities of the original proteins, thus enhancing their overall bioactivity. These proteins are not merely sources of energy but also play pivotal roles in various physiological processes. They exhibit a spectrum of bioactive functions, encompassing antimicrobial, antioxidant, anti-inflammatory, and antitumor properties, thereby underscoring the multifaceted health benefits conferred by whey protein constituents in donkey milk.

The contents for Table 1 are adopted from Baloš et al. (1); Zheleva, (2); Vincenzetti et al. (14); Derdak et al. (43); Cimmino et al. (44); Wang F et al. (45); Li M et al. (46); Claeys et al. (47);; Li Y et al. (48); Garhwal et al. (49); Parsad, (50); Altomonte et al. (51); Martemucci and D’Alessandro, (52).

## 4 Antimicrobial, anti-inflammatory and immunoregulatory effect of donkey milk

### 4.1 Antimicrobial regulatory properties of donkey milk

Recent *in vitro* investigations have elucidated the antibacterial properties of equid milk, attributing these characteristics to the elevated concentrations of lysozyme and lactoferrin (53–55). Furthermore, an association has been observed between the antibacterial activities of donkey milk and its high calcium content (56). The spectrum of anti-bacterial efficacy of donkey milk extends to pathogens such as *Staphylococcus aureus* (57), *S. haemolyticus, Listeria monocytogenes* (58), *Escherichia coli*, and *Salmonella enteritidis* (12, 59, 60). The abundance of lysozyme in donkey milk is particularly noted for its capability to degrade the peptidoglycan layer of Gram-positive bacteria, thus facilitating bacterial lysis (61). Recent research by Saju et al. (36) has demonstrated that endosymbiotic bacteria isolated from donkey milk exhibit pronounced antibacterial activities against *Escherichia coli, S. aureus*, and *Salmonella enterica.* The comprehensive antimicrobial efficacy of donkey milk is likely due to the synergistic interactions among its bioactive components, namely lactoferrin, lysozyme, immunoglobulins, and fatty acids, in addition to anatomical factors related to the udder’s size and position (8, 62). However, a reduction in lysozyme levels during the later stages of lactation has been linked to the presence of pathogenic bacteria in donkey milk (63, 64).

The antimicrobial efficacy of lactoferrin and lysozyme has been substantiated through a compendium of research efforts (18, 65–71). These studies collectively underscore the critical roles that these molecules play in the innate immune defense, particularly through their bacteriostatic and bactericidal activities. Lactoferrin, a multi-functional protein of the transferrin family, is renowned for its ability to sequester iron, thereby inhibiting the growth of iron-dependent bacteria in inflammatory sites. Con-currently, lysozyme, an enzyme that hydrolyzes the bonds in bacterial cell wall peptidoglycan, contributes to the bactericidal activity, particularly against gram-positive organisms. Further investigations have elucidated the synergistic antimicrobial potential when lysozyme and lactoferrin are combined with immunoglobulins and L-amino acid oxidase, as observed in donkey milk (72, 73). This synergism is attributed to the complementary mechanisms of action of these components, where lysozyme and lactoferrin disrupt bacterial cell walls and sequester essential growth nutrients, respectively, while immunoglobulins provide specific immunity and L-amino acid oxidase generates hydrogen peroxide, contributing to an antimicrobial environment.

The bactericidal mechanisms of lysozyme are multifaceted and can be delineated into three principal categories:

Direct bacteriolysis through peptidoglycan disruption: lysozyme exerts a direct bactericidal effect by cleaving the glycosidic bonds within the peptidoglycan layer of bacterial cell walls, a process that is lethal for certain susceptible bacteria. This disruption leads to osmotic imbalance and cell lysis, particularly in some nonpathogenic Gram-positive bacteria such as *Micrococcus luteus* and various Bacillus species. However, this mechanism is not universally effective across all bacterial species, as some exhibit inherent resistance or acquire resistance through structural modifications of peptidoglycan, such as O-acetylation of N-acetylmuramic acid (NAM) and de-N-acetylation of N-acetylglucosamine (NAG), which impede lysozyme’s enzymatic activity.

Indirect antimicrobial effects via enzymatic and non-enzymatic activities: beyond its enzymatic action, lysozyme can exhibit antimicrobial activity through non-enzymatic mechanisms, particularly at elevated concentrations. These activities are attributed to the highly cationic nature of lysozyme, facilitating interactions that can compromise bacterial membrane integrity or induce the release of autolytic enzymes, which are typically involved in bacterial cell wall remodeling. This aspect of lysozyme’s function is evidenced by the retention of antimicrobial activity even when the enzyme’s active site is altered by site-directed mutagenesis, suggesting a mechanism independent of its enzymatic cleavage of peptidoglycan.

Synergistic enhancement with host defense molecules: the bactericidal efficacy of lysozyme, particularly against pathogenic and gram-negative bacteria, is significantly augmented in the presence of other host defense molecules, such as lactoferrin, antibodies, complement proteins, hydrogen peroxide, and ascorbic acid. These cofactors are believed to facilitate the disruption of the outer bacterial membrane, thereby enhancing lysozyme’s access to the peptidoglycan layer. This synergistic interaction underscores the collaborative nature of the innate immune system in combating bacterial infections. Furthermore, recent studies have illuminated lysozyme’s role in modulating the host immune response. The enzymatic degradation and subsequent lysis of bacteria by lysozyme result in the release of bacterial components, including peptidoglycan fragments, which can activate pattern recognition receptors on host cells. This process not only contributes to the immediate defense against bacterial invasion but also plays a pivotal role in the activation and regulation of broader immune responses (18, 74–76). Thus, lysozyme’s contribution to antimicrobial defense extends beyond direct bactericidal activity, encompassing roles in immune modulation and synergy with other antimicrobial pathways.

**Figure 2:**
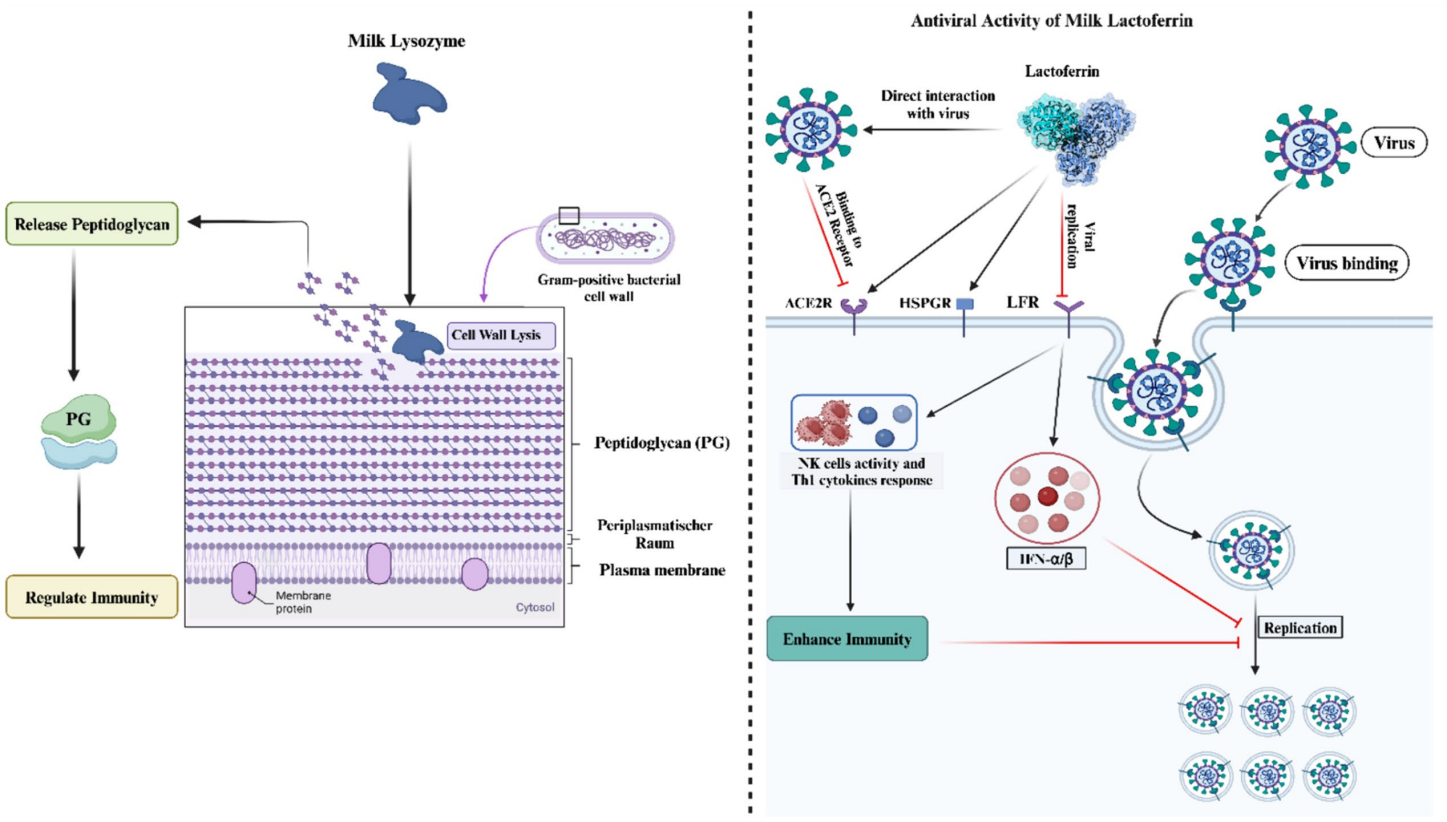
The antimicrobial properties of donkey milk.

Moreover, lactoferrin has demonstrated significant antiviral activity, inhibiting viral attachment to host cells and replication within them. This is achieved through the blockade of key viral receptors [Angiotensin-converting enzyme 2 (ACE), Heparan sulfate proteoglycan (HSPG)] and the induction of antiviral cytokines such as interferon (IFN)-*α*/*β* (77, 78). The administration of lactoferrin has also been shown to enhance natural killer (NK) cell activity and Th1 cytokine responses, thereby bolstering defenses against viral pathogens (79). The antimicrobial mechanism of donkey milk has been presented in Figure 2.

### 4.2 Anti-inflammatory and immunoregulatory role of donkey milk

The anti-inflammatory and immunomodulatory effects of donkey milk have garnered increasing scientific interest, as evidenced by recent studies (Table 2). Consistently, Kocyigit et al. (80) provided a foundational exploration into the anti-inflammatory properties of equid milk, setting a precedent for subsequent investigations in this domain. Our research team further substantiated these findings, demonstrating that the anti-inflammatory and antioxidative attributes of donkey milk contribute significantly to its regulatory effects on health (48). In a detailed study, Taghiloo et al. (81) elucidated the impact of donkey milk on immune cell function, revealing that treatment with donkey milk resulted in elevated levels of interleukin-8 (IL-8) and interleukin-6 (IL-6) in peripheral blood mononuclear cells (PBMCs). IL-8 serves as a chemotactic factor for neutrophils, while IL-6 plays a crucial role as an acute phase protein with protective functions. Furthermore, donkey milk was found to modestly enhance the production of tumor necrosis factor-alpha (TNF-*α*) from PBMCs, indicating a relatively low pro-inflammatory profile. The stimulation of normal human PBMCs with donkey milk also led to the release of interleukin-10 (IL-10) in supernatants, highlighting its potential to modulate immune responses and maintain immune homeostasis (81). These cytokines are instrumental in the maturation, differentiation, and regulation of immune cells, thereby facilitating the host’s defense mechanisms (82). Previous investigations have shown that donkey milk can induce the production of nitric oxide (NO) from PBMCs and promote adaptive immunity through cytokine production (83). In a mouse model of ileitis, donkey milk exhibited anti-inflammatory properties, which were associated with the normalization of antimicrobial peptides levels (lysozyme and *α*-defensin) in Paneth cells and a reduction in the dysbiosis typically associated with ileitis (84). Additionally, the impact of donkey milk on gut barrier function was observed in mice subjected to water-avoidance stress, further underscoring its therapeutic potential (85).

**Table 2:** Antimicrobial, anti-inflammatory, antioxidant and immune regulatory role of donkey milk.

References	Donkey milk associated biological effect
Zheleva (2)	Anti-inflammatory, immunoregulatory, antioxidant and antimicrobial potential
Li et al. (3)	Antioxidant properties and antiallergic properties
Aspri et al. (13)	ACE-inhibitory activity
Akan (27)	Antioxidant and antidiabetic potential
Tafaro et al. (28)	Immunoregulatory properties
Saju et al. (36)	Antibacterial potential of donkey milk endosymbiotic bacteria against *Escherichia coli, Staphylococcus aureus* and *Salmonella enterica*
Spada et al. (72)	Antibacterial properties of Donkey milk
Li et al. (48)	Antioxidant and anti-inflammatory properties
Taghiloo et al. (81)	Immunoregulatory effect
Jirillo and Magrone (82)	Anti-inflammatory and antiallergic properties
Yvon et al. (85)	Anti-inflammatory and antimicrobial potential
Garhwal et al. (49)	Antimicrobial, anti-inflammation, antioxidant, and hypo-allergenicity properties
Beghelli et al. (164)	Antioxidant properties
Bhardwaj et al. (165)	Antioxidant properties
Simos et al. (116)	Antioxidant and anti-platelet properties
Trinchese et al. (147)	Improved antioxidant efficiency and anti-inflammatory status

The anti-inflammatory capabilities of donkey milk have also been linked to the prevention of allergic asthma, showcasing its broad spectrum of health benefits (86). Moreover, a recent study by Farias et al. (87) demonstrated that asinine milk significantly downregulated salivary cortisol levels and the expression of the IL-1B gene in weaning piglets, thereby mitigating stress-induced inflammatory responses. Collectively, these studies highlight the multifaceted role of donkey milk in modulating inflammation and immune responses, suggesting its potential as a therapeutic agent in various inflammatory and autoimmune conditions.

## 5 Unlocking the health benefits of donkey milk: *in vitro* insights

### 5.1 Anticancer activity of donkey milk

Donkey milk is recognized as a reservoir of numerous salutary components, including caseins, Omega-3 fatty acids, lactoferrin, lysozyme, *α*-lactalbumin, and *β*-lactoglobulin, which contribute to its potential therapeutic applications. The inclusion of long-chain omega-3 polyunsaturated fatty acids (PUFAs) is of particular interest due to their documented role in the prophylaxis of various carcinomas, including but not limited to breast and colorectal cancers (88–97). Consistently, research delineates the inhibitory effects of *α*-casein on the activity of breast cancer stem cells and cancer-associated fibroblasts in MDA-MB-231 cells, mediated through the modulation of HIF-1*α*, STAT3, and STAT19 expression (98). Concurrently, Maurmayr et al. have elucidated the potent anti-tumorigenic properties of lactoferrin, α-lactalbumin, and *β*-lactoglobulin against a spectrum of human tumor cell lines, including A549, HT29, HepG2, and MDA231-LM2 (99).

Investigations into the bioactivity of donkey milk on A549 human lung cancer cells have revealed its capacity to augment the secretion of a cadre of cytokines, including IL-2, IFN-*γ*, IL-6, TNF-α, and IL-1β, thereby indicating a dual mechanism of action: direct antiproliferative effects on tumor cells and indirect tumoricidal effects mediated through the activation of lymphocytes and macrophages (100). This assertion is bolstered by Shariatikia et al. (101), who reported dose-dependent cytotoxic effects of donkey milk casein and whey proteins against MCF7 cells. The antiproliferative, antimutagenic, and antibacterial properties of donkey milk kefir have also been documented, with evidence suggesting its role in inducing apoptosis, inhibiting cell proliferation, and reducing the co-expression of iNOS and endothelial-NOS in the context of Ehrlich ascites carcinoma (EAC) in mice (102–105).

Moreover, the comparative analysis of donkey colostrum (DC) and mature milk (DM) on 4 T1 triple-negative breast cancer (TNBC) tumors in mice has yielded promising outcomes, with both DC and DM significantly attenuating the primary tumor volume and the relative organ weight of the liver and lungs in the 4 T1 mouse model, without adverse effects on body weight. This antitumor efficacy is attributed to the modulation of apoptotic and angiogenic markers, including an increase in cleaved-caspase-3 and Bax expression and a decrease in MMP2 and CD31 levels (26). Additionally, Akca et al. (106) have identified the anti-proliferative and genotoxic effects of donkey milk on lung cancer cells, with a specificity that spares normal lung cells, further underscoring the potential of donkey milk as a functional food with therapeutic implications in oncology.

### 5.2 Antidiabetics potential of donkey milk

Bioactive peptides, derived from a variety of protein sources with a notable emphasis on milk proteins, are increasingly recognized for their multifaceted biological functionalities. These include, but are not limited to, antioxidant, antimicrobial, anti-diabetic, antihypertensive, anticancer, and antitumor properties (107, 108). Type II diabetes mellitus, also referred to as non-insulin dependent diabetes, emerges as a pre-dominant metabolic disorder characterized by persistent hyperglycemia, with a significant global incidence annually (27, 109). The etiology of Type II diabetes is multi-factorial, with obesity, *β*-cell dysfunction, and insulin resistance in peripheral tissues being primary contributors (110–112).

A pivotal aspect of Type II diabetes management involves the modulation of di-peptidyl peptidase-IV (DPP-IV) and *α*-glucosidase activities, enzymes integral to the inactivation of incretins that are crucial for normoglycemic regulation (113, 114). The inhibition of these enzymes constitutes a strategic approach in the therapeutic management of this condition. Empirical evidence from *in vivo* investigations has under-scored the regulatory influence of food proteins on serum glucose concentrations (115). Corroborating these findings, *in vitro* studies have demonstrated that peptides derived from casein significantly attenuate the activity of DPP-IV and *α*-glucosidase, while concurrently enhancing antioxidant capacities, thereby contributing to the management of Type II diabetes (27).

Furthermore, the antidiabetic potential of donkey milk has been substantiated through various research endeavors (2, 42, 116, 117). Consequently, Li et al. (117) elucidated that donkey milk not only ameliorates the viability of compromised pancreatic *β* cells but also enhances insulin sensitivity in target organs without directly stimulating insulin secretion from the damaged β cells. This is attributed to the presence of *α*-lactalbumin in donkey milk. Additionally, donkey milk has been shown to decrease glycosylated hemoglobin levels and exert a therapeutic effect on diabetes by downregulating the expression of key hepatic gluconeogenesis enzymes, namely phosphoenolpyruvate carboxykinase 1 and glucose-6-phosphatase (117). Collectively, these studies highlight the significant potential of bioactive peptides, particularly those derived from milk proteins, in the prevention and management of type II diabetes through various biochemical mechanisms. The key findings of studies reported the role of donkey milk in health improvement have been summarized in Table 3.

**Table 3:** Donkey milk role in human health.

References	Health linked bioactive components in donkey milk	Health benefit
Li et al. (26)	Milk α-casein, lactoferrin, α-lactalbumin, and β-lactoglobulin	Triple-negative breast cancer
Akan (27)	milk casein	α-glucosidase inhibitor and Antidiabetic
Tafaro et al. (28)	Rich in omega-3 fatty acids	Donkey colostrum and milk induced nitric oxide (NO) release from PBMCs
Yvon et al. (85)	lysozyme	Used in treatment of ileitis
Lu et al. (86)	Donkey milk powder (15% protein, 64% carbohydrate and 6% fat, including 8% whey protein and 0.5% polyunsaturated fatty acid)	Alleviated ovalbumin-induced asthma Relieved airway hyperresponsiveness, injury and fibrosis of airway epithelium Reduced airway eosinophilia and the increased Th2 cytokines in bronchoalveolar lavage fluid as well as serum immunoglobulin E, and inhibited NF-κB P65 activity.
Mao et al. (100)	Milk lysozyme	Prevent lung cancer
Shariatikia et al. (101)	Casein and whey proteins	Prevent Breast cancer
Esener et al. (104)	milk kefir	Ehrlich ascites carcinoma
Khan et al. (107)	Milk lactoferrin and lysozyme	Cytotoxic and genotoxic on human
Trinchese et al. (166)	–	Improved heart mitochondrial metabolic flexibility and antioxidant potential Prevent hypertension and heart failure
Tang et al. (167)	Donkey milk protein	Enhanced the gastrointestinal function and reduce the incidence of gastrointestinal motility disorder in patients with severe pneumonia
Kocic et al.; Li et al. (168, 169)	Donkey milk	Enhance the skin fibroblast survival and their proliferative and regenerative potential Restored skin barrier by upregulating the level of filaggrin Enhance wound healing

### 5.3 Use of donkey milk in cow’s milk protein allergy children

**Figure 3:**
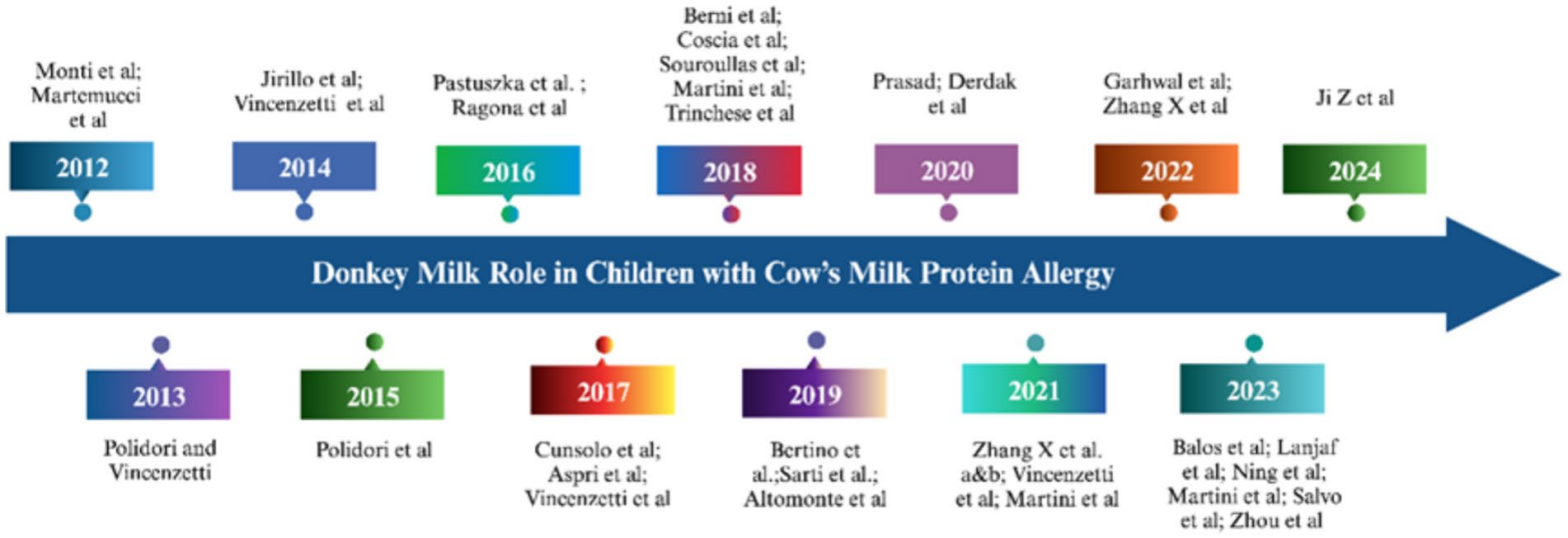
Research development of donkey milk as alternative in infants with milk cow allergy (1, 6, 7, 12–14, 38, 43, 50–52, 63, 82, 124, 147–163).

The research development on donkey milk as alternative for infants with cow milk allergy has been summarized in Figure 3. Cow’s Milk Protein Allergy (CMPA) delineates an aberrant immunologic response toward proteins presents in cow’s milk, manifesting in a subset of individuals. As a prevalent food allergy, particularly in developed nations, cow’s milk proteins often represent the initial exogenous proteins introduced to infants, with an incidence rate of 2–7% in children under 6 months, which notably diminishes to 0.1–0.5% in adults (118, 119). The allergic manifestations to cow’s milk can be attributed to diverse immunological pathways, including immediate IgE-mediated hypersensitivity, characterized by symptom onset within 30 min post-ingestion, and delayed non-IgE mediated reactions, where symptoms emerge hours to days subsequent to consumption (120, 121). The main allergens in cow’s milk are primarily the caseins (*α*-s1- and *β*-caseins). However, β-lactoglobulin and α-lactalbumin are also involved to a lesser extent (118). It is worth noting that the lower allergenic potential of certain types of milk, such as that from non-ruminant animals like donkeys or horses, is not only due to their lower casein content but also to significant sequence differences between the proteins in their milk and those found in ruminants’ milk. Additionally, the reduced allergenicity observed in non-ruminant milk may largely be a result of substantial sequence differences between β-lactoglobulin in non-ruminants and its homologous protein in ruminants.

Addressing CMPA necessitates the comprehensive exclusion of cow’s milk from the diet. Given the critical role of milk as a nutritional mainstay up to the age of two, outright elimination is inadvisable; instead, alternative milk sources with appropriate nutritional profiles, low allergenic potential, palatability, and economic viability are recommended. While hypoallergenic formulas are preferred for managing CMPA, the incorporation of milk from other species warrants careful pediatric oversight. The diminished allergenicity of donkey milk is chiefly ascribed to its lower casein content (42, 122). Moreover, the high whey protein ratio in donkey milk facilitates easier digestion (123). Despite its lower fat and caloric density, the nutritional value of donkey milk can be enhanced through the addition of medium-chain triglycerides (8). This fat supplementation strategy is equally applicable to lyophilized donkey milk, which according to Vincenzetti et al. (42), retains its nutritional integrity in comparison to its raw counterpart.

## 6 Factors compromising the compositional properties and quality of donkey milk

The compositional properties and quality of donkey milk are influenced by a multifaceted array of factors, which include but are not limited to the method of preservation, the stage of lactation, and dietary considerations. These elements have been identified as pivotal in determining the nutritional and biochemical profile of donkey milk, with implications for both its quality and utility in various applications (124–126). The factors affecting quality and quantity of donkey milk are summarized in Table 4.

**Table 4:** Effect of lactation stage, diet and method of preservation/treatment on donkey milk production traits.

Biological factors	Effect on donkey milk production traits	References
Diet effect		
selenium yeast supplementation (0.3 mg/kg)	Improved milk components and lactational performance	(142)
Dietary roughage supplementation	Improved milk lipid molecules and volatile organic compounds (VOCs)	(145)
Crude protein (14.2%)for 10 weeks	Enhanced milk yield and yields of protein, lactose, solid-not-fat, total solid, and contents of protein, total solid and milk urea nitrogen	(170)
Concentrate to forage feed ratio (30:70) 8 weeks	Increased milk protein and most amino acid (AA) production in milk	(171)
Lactation stage		
	Altered protein profile	(63)
	Alteration in milk microbiota	(64)
	Changes in milk mineral contents (cupper and selenium concentrations decreased; manganese increased)	(172)
Treatment/preservation effect		
HTST plus HPP treatment at 300 MPa HTST plus HPP treatment at 400 MPa HTST plus HPP treatment at 300 MPa	Maintained the milk lysozyme, α-lactalbumin and *β*-lactoglobulin contents in donkey milk. Reduced Donkey milk stability Promoted shelf life of donkey milk	(32, 173)
Combination of pasteurization and HPP (600 MPa/180 s and 400 MPa/180 s)	Compromised the quality of donkey milk Unfit for human consumption	(173)
UHPH at 100 MPa	Maintained the minimum shelf-life of donkey milk for 28 days. Did not affect the level of milk lysozyme	(174)
Freeze drying method	Preserved the bioactive components and overall shelf life of donkey milk Reduced the transport and the storage costs of donkey milk	(175, 176)

HHP, High hydrostatic pressure; HTST, high-temperature short time pasteurization; HHP, high hydrostatic pressure; UHPH, Ultra-high-pressure homogenization.

### 6.1 Lactation stage and its impact on donkey milk composition and quality

The stage of lactation has been observed to exert a significant influence on the compositional attributes of donkey milk, particularly with respect to its protein, lipids and volatiles contents (127). Research indicates a progressive decline in milk protein concentration as the lactation period extends (128, 129). This trend is corroborated by Malacarne et al. (40), who noted a discernible impact of the lactation phase on the protein and mineral content of donkey milk, albeit with caseins being an exception. Contrarily, Zhou et al. (63, 64) documented a fluctuating pattern in the relative expression levels of various casein proteins, which initially increased before diminishing as lactation progressed. Complementing these findings, dos Santos et al. (130) reported a decline in total solids and fats in correlation with advanced lactation stages, whereas Salgado et al. (131) observed an enhancement in total polyunsaturated and n − 3 fatty acids over time. Intriguingly, aging was linked with an elevation in protein and fat concentrations in milk, alongside a notable impact on the principal whey proteins, whose contents were also modulated by the lactation stage (130). The expression levels of lactoferrin and other whey proteins such as albumin, lysozyme, *β*-lactoglobulin, and *α*-lactalbumin exhibit-ed dynamic changes during the lactation period, with a significant portion of donkey milk protein being constituted by whey proteins (40). The lipid profile of donkey milk was also found to be significantly altered by lactation, with hormonal shifts, particularly in prolactin levels, being implicated in the observed reduction in lipid content (132, 133).

### 6.2 Dietary influences on donkey milk production traits

Nutritional strategies play a crucial role in optimizing the health and productive performance of livestock, including lactating donkeys. There is a substantial body of evidence underscoring the positive effects of nutrition on the overall well-being and output of livestock animals (134–141). Recent investigations have highlighted the potential of nutritional management during lactation to mitigate oxidative stress and enhance the health and lactational efficiency of donkeys (142–144). Specifically, dietary supplementation with selenium yeast (0.3 mg/kg) has been shown to significantly improve the composition and quality of donkey milk, as well as the overall lactational performance (142). Furthermore, the inclusion of dietary roughage has been associated with an upregulation of lipid molecules and volatile organic compounds in donkey milk, indicating the profound impact of diet on milk compositional traits (145).

### 6.3 Effect of breed on milk production traits in donkeys

The variability in donkey milk production and composition is closely linked to the specific breed of the animals involved. Studies have demonstrated that certain breeds exhibit markedly higher milk yields compared to others. For instance, Istrian donkeys have been shown to produce significantly more milk than Littoral Dinaric donkeys (146). Similarly, higher milk yields were observed in the Martina Franca and Ragusana breeds when compared with the Amiata jennies (129). Beyond yield, there are notable differences in the compositional traits of milk across various donkey breeds (40, 58, 144, 146). These findings underscore the critical influence of breed as a determining factor in both the quantity and quality of milk produced by donkeys. The breed-specific differences in milk composition likely reflect variations in genetics, physiology, and lactation dynamics, emphasizing the importance of considering breed as a primary factor in the selection of donkeys for milk production purposes. These findings collectively underscore the intricate interplay between lactation stage, dietary practices, and preservation/treatment methods in shaping the compositional quality of donkey milk. Understanding these dynamics is essential for devising strategies to optimize the nutritional value and functional properties of donkey milk for various applications.

## 7 Future perspectives and limitations

Future studies could focus on the in-depth analysis of the bioactive compounds present in donkey milk, such as lysozyme, lactoferrin, and immunoglobulins, which contribute to its antimicrobial and immunomodulatory activities. The exploration of these components could lead to the development of functional foods or supplements aimed at enhancing immune function and overall health. Rigorous clinical trials are necessary to substantiate the health claims associated with donkey milk. This includes its purported benefits in managing allergies, improving gut health, and supporting the immune system. Establishing a strong evidence base will be crucial for gaining regulatory approval and consumer trust in donkey milk as a health-promoting food. As interest in donkey milk increases, sustainable and ethical farming practices must be developed to meet demand without compromising animal welfare or environmental integrity. This includes addressing the challenges of low milk yield and seasonal lactation in donkeys through advancements in animal husbandry and dairy technology. Efforts should be made to increase consumer awareness and acceptance of donkey milk as a nutritious and health-promoting food. This involves overcoming cultural and perceptual barriers, as well as establishing a premium market segment for donkey milk products, akin to those for other non-conventional dairy products like goat or sheep milk.

One of the primary limitations in the use of donkey milk is its low production yield. Donkeys produce significantly less milk than cows, goats, or sheep, and the milking process can be labor-intensive, contributing to higher production costs and limited availability. There is currently a lack of standardization in the production and processing of donkey milk, which can lead to variability in product quality and nutritional content. Establishing industry standards and regulatory guidelines will be essential for ensuring consistency and safety in donkey milk products. Despite promising preliminary research, there are still significant gaps in our understanding of the health benefits and nutritional properties of donkey milk. More comprehensive studies are needed to elucidate its mechanisms of action and potential therapeutic applications. The economic viability of donkey milk production on a large scale is questionable due to the aforementioned production challenges and market acceptance issues. Developing cost-effective production methods and creating value-added products could help mitigate these concerns.

## 8 Conclusion

In conclusion, donkey milk presents an intriguing option for health-promoting foods, with its unique nutritional profile and potential therapeutic properties. However, still need deep studies involving both invitro and *in vivo* experiments to establish the molecular mechanism through which donkey milk showed therapeutic effect against several diseases like diabetes, cardiovascular diseases, asthma, hypoallergy and various types of cancers. In addition, realizing its potential will require overcoming significant challenges related to production, research, and market development. Future studies and innovations in dairy technology will play a critical role in addressing these limitations and unlocking the potential of donkey milk as a valuable addition to the functional food market.
